# Network analysis of spousal support and fear of childbirth in pregnant women of advanced maternal age

**DOI:** 10.3389/fpsyt.2026.1848470

**Published:** 2026-06-22

**Authors:** Lili Zhu, Fandi Yin, Jing Li, Xue Cheng, Yuhan Li, Yiwen Cai, Fan Fan, Xiaoxia Chen

**Affiliations:** 1 School of Nursing, Henan Medical University, Xinxiang, China; 2 School of Nursing, Soochow University, Suzhou, China; 3 Teaching and Administration Department, Xinxiang First People’s Hospital, Xinxiang, China

**Keywords:** advanced maternal age, fear of childbirth, network analysis, perinatal mental health, spousal support

## Abstract

**Background:**

Fear of childbirth is an important perinatal mental health concern, particularly among women of advanced maternal age. However, the specific interrelations between spousal support and fear of childbirth remain unclear.

**Methods:**

This cross-sectional study recruited 279 pregnant women of advanced maternal age from a tertiary hospital in Henan, China, using convenience sampling. Spousal support and fear of childbirth were assessed using the Spouse Support Inventory and the Childbirth Attitude Questionnaire. A regularized partial-correlation network was estimated using EBICglasso, and central and bridge nodes were identified. Network stability was examined using bootstrap procedures.

**Results:**

The prevalence of any fear of childbirth, defined as a CAQ score ≥28 and including mild, moderate, and severe categories, was 86.4% (n = 241). Negative associations predominated between the spousal support and fear of childbirth communities. The strongest cross-community association was observed between “teaching you how to do things you do not know how to do” and “concern about fetal health.” The most central nodes were “participating in activities together to reduce your stress” and “providing you with helpful information,” whereas the strongest bridge nodes were “helping you understand why things did not go well” and “giving you encouragement.

**Conclusion:**

Specific supportive behaviors, especially informational and cognitive-appraisal support, occupied central positions in the network linking spousal support and fear of childbirth among pregnant women of advanced maternal age. Strengthening these forms of spousal support may inform the development of couple-based interventions to reduce childbirth fear and promote perinatal mental health.

## 1 Introduction

Against the backdrop of persistently declining birth rates and generally low fertility intentions among women in China, improving childbirth experiences to facilitate the implementation of pro-fertility policies has become a major societal concern (1). Childbirth is not just a physiological process; it is profoundly influenced by psychological, social, and cultural factors. Since the implementation of China’s universal two-child and three-child policies, the demographic characteristics of pregnant women have undergone significant changes. Regional surveys have shown that the proportion of pregnant women of advanced maternal age (AMA) increased from 8.52% before the policy change to 15.82% (2). In certain tertiary hospitals, this proportion now exceeds 20% (3). The increasing prevalence of advanced maternal age presents significant challenges for antenatal psychological care.

This shift in demographics brings an increased likelihood of obstetric complications. Women of advanced maternal age are more susceptible to conditions such as gestational hypertension, gestational diabetes, and placental abnormalities, which can heighten anxiety and fear of childbirth (FOC) (4, 5). Epidemiological studies show that the prevalence of FOC differs widely across countries and regions. A recent study summarized that reported prevalence rates ranged from 12% to 58.6%, depending on cultural context, healthcare availability, and the tools used for assessment (4). Due to increased concerns about pregnancy and fetal health, women of advanced maternal age tend to report higher levels of FOC (5). A multicenter prospective study found that the risk of adverse pregnancy outcomes rises nonlinearly with maternal age, with an inflection point between 35.6 and 40.4 years (6). Previous research has also linked fear of childbirth to cesarean birth (7). Notably, among women of very advanced maternal age (≥45 years), the cesarean rate may reach 84.4% (6). Taken together, these results suggest that psychological stress in this group may influence mode-of-childbirth decisions and dampen subsequent fertility intentions (1). This is at odds with the World Health Organization’s goal of promoting a positive childbirth experience (8).

Social support may ease the psychological burden of women of AMA throughout pregnancy and childbirth. Spousal support is one of the most immediate and stable forms of family support and may buffer pregnancy-related stress (9–12). Spousal support encompasses both emotional support, including comfort, understanding, and encouragement, and practical help, such as sharing antenatal information and providing day-to-day care (9–12). Studies further suggest that limited social support, particularly from partners and family members, is associated with FOC and adverse emotional states during pregnancy, including anxiety and depression (9, 10). A recent systematic review and meta-analysis further reported significant inverse associations between FOC and both perceived social support and partner support, whereas family and friend support showed no significant associations with FOC, highlighting the particular relevance of partner-related support in childbirth fear research (13). Relationship quality may also be an important relational resource related to fear of childbirth (FOC). In a sample of Italian pregnant women, higher partner relationship quality was associated with lower FOC, whereas insufficient partner support was linked to a higher risk of FOC (11). Research in pregnant adolescents has reported a significant inverse association between social support, particularly spousal support, and FOC, suggesting that low spousal support may be a modifiable target for intervention in high-risk populations (12). In women of AMA, enhancing spousal support may likewise help improve psychological well-being and support a positive childbirth experience (9–12).

However, existing studies have predominantly relied on traditional sum-score or latent variable models, which typically examine linear associations between spousal support and FOC only at an aggregate level and may fail to capture complex item-level interactions (14, 15). Consequently, it remains unclear which supportive behaviors may be more relevant to specific dimensions of childbirth fear, such as pain-related concerns or loss of control. To address these methodological gaps, network analysis has been increasingly used in psychopathology research (16). It offers a useful way to examine item-level associations among variables. In addition, it can identify central nodes and bridge nodes linking distinct psychological communities, thereby highlighting potential intervention targets and pathways (17).

Network analysis has been widely used in studies of anxiety, depression, symptom clusters, and quality of life (18, 19). However, little is known about the network associations between FOC and spousal support in women of AMA. In this study, we estimated a regularized partial-correlation network of spousal support and FOC within a Gaussian graphical model. We aimed to examine how these variables were connected, identify central nodes and bridge pathways, and consider the findings from the perspectives of dyadic coping and emotion regulation. Ultimately, the results may help inform the development of targeted, couple-based interventions.

## 2 Materials and methods

### 2.1 Study design and participants

From March to October 2025, we conducted a cross-sectional study among women of advanced maternal age. Participants were recruited by convenience sampling from the obstetrics outpatient clinic of a tertiary Grade A hospital in Xinxiang, Henan Province, China. The inclusion criteria were as follows: (1) age 35 years or older; (2) singleton pregnancy; (3) gestational age of at least 28 weeks; (4) registration at the study hospital with regular antenatal care; (5) ability to understand and complete the questionnaire independently; and (6) provided written informed consent. Exclusion criteria were as follows: (1) severe fetal developmental anomalies or intrauterine fetal death confirmed by prenatal diagnosis; (2) clinically diagnosed major psychiatric disorders or significant cognitive impairment; (3) severe cardiac, hepatic, or renal insufficiency preventing continuation of pregnancy or completion of the assessment; and (4) active labor, premature rupture of membranes, or unstable maternal or fetal conditions preventing participation in the survey. For sample-size planning, the network comprised 22 nodes. Following a parameter-counting approach for network models, we considered 22 node-level parameters and 231 pairwise association parameters [22×(22−1)/2], yielding 253 parameters in total, which served as a preliminary reference for recruitment planning. Assuming an invalid-response rate of approximately 15%, 298 questionnaires were planned for distribution. Of the 298 questionnaires distributed, 279 were valid, yielding a valid response rate of 93.62%.

### 2.2 Measures

#### 2.2.1 General demographic and clinical information questionnaire

A researcher-developed questionnaire, based on a literature review, was used to collect participants’ characteristics. The questionnaire included two sections: (1) Socio-demographic characteristics: age, occupation, educational level, monthly per capita household income, place of residence, and method of payment for medical expenses; (2) Obstetric and clinical data: gestational age, parity, mode of conception, whether the current pregnancy was planned, history of adverse pregnancy outcomes, pregnancy-related comorbidities and complications, history of pregnancy-prolonging treatment, planned mode of childbirth, and intention to use labor analgesia.

#### 2.2.2 Childbirth attitude questionnaire

Developed by Lowe (20) based on the FCQ and subsequent related research, and culturally adapted for Chinese populations by Zhang (21), the CAQ was used to assess antenatal FOC. The 16-item scale assesses FOC across four domains: fetal well-being, labor pain and injury, self-control, and medical care. Items are rated on a 4-point Likert scale from 1 (none) to 4 (severe), producing a total score of 16–64; higher scores indicate greater FOC. According to commonly used CAQ classification criteria in Chinese pregnant populations, total scores of 16–27 indicate no FOC, 28–39 indicate mild FOC, 40–51 indicate moderate FOC, and 52–64 indicate severe FOC. In the present study, the overall prevalence of any FOC was defined as the proportion of participants with a CAQ score ≥28, including mild, moderate, and severe FOC categories, consistent with prior CAQ-based studies in Chinese pregnant populations (5, 22). Cronbach’s α was 0.882 for the Chinese version (21) and 0.780 in the present study.

#### 2.2.3 Spouse support inventory

The SSI, developed by Zhang et al. (23), was administered to measure support received within the marital relationship. It contains 18 items covering two domains, subjective support and objective support. Items are scored on a 7-point Likert scale from 1 (never) to 7 (always), giving a total score of 18–126, with higher scores reflecting greater support. Cronbach’s α was 0.960 for the original scale (23) and 0.955 in the present study.

### 2.3 Data collection and quality control

Before data collection, two researchers received standardized training on the questionnaires, participant instructions, and field procedures to ensure consistency. Approval was obtained from hospital management and the relevant department heads, after which data were collected in the waiting area of the antenatal clinic. Women in the third trimester were screened for eligibility and invited to take part. After the study purpose, confidentiality, and voluntary participation were explained, written informed consent was obtained. Paper questionnaires were then completed with researcher support, typically within 15–20 minutes. When a participant had difficulty reading or writing, the researcher read each item aloud in a neutral manner and recorded the responses. Questionnaires were checked immediately after completion, and any missing items were completed on site.

### 2.4 Statistical analysis

Analyses were performed in SPSS 26.0 and R 4.5.2. Categorical data are reported as counts and percentages, and continuous variables with a normal distribution are summarized as mean ± standard deviation (SD). To provide clinical context, descriptive and stratified analyses were conducted to examine the prevalence of any FOC and CAQ total scores across selected obstetric and clinical characteristics. Group differences in CAQ total scores were assessed using t-tests or one-way ANOVA, as appropriate.

Network analysis was conducted in R using the qgraph, bootnet, networktools, and mgm packages. Following the methodological recommendations of Epskamp et al. (15) and the mixed-node strategy described by Zhou et al. (24), a 22-node network was constructed, including 4 nodes from the CAQ and 18 nodes from the SSI. In the primary network, the 18 items of the Spouse Support Inventory were included as item-level nodes to capture specific supportive behaviors, whereas the Childbirth Attitude Questionnaire was represented by its four theoretically defined domains to preserve the conceptual structure of fear of childbirth and avoid excessive fragmentation of this construct, and maintain an adequate ratio of sample size to estimated parameters for stable network estimation. For the CAQ domains, mean domain scores were used rather than raw summed scores to reduce score-range differences caused by unequal numbers of items across domains. Within the Gaussian graphical model framework, the correlation matrix was estimated using the cor_auto function to accommodate mixed variable types. A regularized partial-correlation network was then estimated using EBICglasso with a tuning parameter of γ = 0.5. The Fruchterman–Reingold layout was used to visualize the network, placing more strongly connected nodes closer together. Node centrality was assessed using expected influence (EI), with higher EI values indicating greater overall influence within the network. To identify bridging pathways between fear and support systems, nodes were assigned *a priori* to three communities according to the theoretical structure of the measures and the study aim: FOC, subjective support, and objective support. The four CAQ domain nodes were grouped into the FOC community, whereas SSI nodes were grouped into subjective-support and objective-support communities according to the SSI subscales. This community assignment was based on predefined scale structure rather than data-driven clustering, which warrants a cautious interpretation as this *a priori* configuration may influence the bridge centrality estimates. Bridge expected influence (BEI) was then calculated using the networktools package. Node predictability was computed using the mgm package. Network stability and accuracy were examined using bootstrapping in the bootnet package. First, the stability of centrality indices was assessed using case-dropping bootstrap and quantified by the correlation stability (CS) coefficient; a CS coefficient ≥ 0.50 is generally considered indicative of good stability (15). Second, the accuracy of edge-weight estimation was evaluated by non-parametric bootstrap 95% confidence intervals (CIs), with narrower CIs indicating higher precision. To examine whether the main findings were robust to the mixed-granularity node specification, we conducted a dimension-level sensitivity analysis. In this supplementary 6-node network, spousal support was represented by the subjective and objective support dimensions, whereas fear of childbirth was represented by the four CAQ domains (see **Supplementary Figure 4**).

### 2.5 Ethical considerations

This study was approved by the Ethics Committee of Xinxiang Central Hospital, Henan Province, China (Approval No. 2024-192). Written informed consent was obtained from all participants before data collection. Participation was voluntary, and participants were assured of anonymity and confidentiality.

## 3 Results

### 3.1 Participant characteristics

The sample comprised 279 pregnant women of advanced maternal age. As shown in **Table 1**, 134 participants (48.0%) had a junior college education, and 175 (62.7%) were currently employed. Regarding childbirth preferences, 249 participants (89.2%) reported an intention to use labor analgesia, and 161 (57.7%) preferred vaginal birth. In addition, 84 participants (30.1%) reported pregnancy-related comorbidities.

**Table 1:** Participant characteristics (n = 279).

Variables	Variable categories	n	%
Age (years)	35-36	187	67.0
	37-39	70	25.1
	≥40	22	7.9
Education	Senior high school or below	43	15.4
	Junior college	134	48.0
	Bachelor’s degree or above	102	36.6
Occupation	Employed	175	62.7
	Unemployed	104	37.3
Monthly household income per capita (CNY)	<5000	104	37.3
	5000-7999	110	39.4
	>8000	65	23.3
Current residence	Urban	194	69.5
	Township	36	12.9
	Rural	49	17.6
Method of payment for maternity care	Medical insurance	213	76.3
	Self-pay	66	23.7
Current gestational age (weeks)	28-32	37	13.3
	33-36	169	60.6
	≥37	73	26.2
Parity	Nulliparous	150	53.8
	Multiparous	129	46.2
Planned pregnancy	Yes	191	68.5
	No	88	31.5
History of adverse pregnancy outcomes	Yes	70	25.1
	No	209	74.9
Use of tocolytic medication during pregnancy	Yes	93	33.3
	No	186	66.7
Preferred mode of childbirth	Vaginal birth	161	57.7
	Cesarean section	47	16.8
	No strong preference	71	25.4
Intention to use labor analgesia	Yes	249	89.2
	No	30	10.8
Pregnancy comorbidities or complications	Yes	84	30.1
	No	195	69.9

### 3.2 Levels of FOC and spousal support

In the 279 women of advanced maternal age, the mean FOC score was 38.51 ± 9.97. Using a CAQ score ≥28 as the threshold for any FOC, 241 participants (86.4%) were classified as having FOC. This prevalence included mild, moderate, and severe categories: 111 participants (39.8%) had mild FOC, 104 (37.3%) had moderate FOC, and 26 (9.3%) had severe FOC (**Table 2**). The mean total spousal support score was 80.89 ± 22.60, and the mean subjective and objective support scores were 40.53 ± 11.46 and 40.36 ± 11.58, respectively (**Table 3**). Stratified analyses of the prevalence of any FOC and CAQ total scores across selected obstetric and clinical characteristics are presented in **Supplementary Table 3**. CAQ total scores differed significantly by parity (*P* = 0.028) and history of adverse pregnancy outcomes (*P* = 0.032), whereas no significant differences were observed for the other selected characteristics.

**Table 2:** Severity classification of FOC based on CAQ cut-off scores among pregnant women of advanced maternal age (n = 279).

FOC level	Criteria	n	%
No FOC	16-27	38	13.6
Mild FOC	28-39	111	39.8
Moderate FOC	40-51	104	37.3
Severe FOC	52-64	26	9.3

**Table 3:** Total and subscale scores for spousal support among pregnant women of advanced maternal age (n = 279).

Dimension	Score range	Total score	Mean item score
Spousal support	18-126	80.89 ± 22.60	4.49 ± 1.26
Subjective support	9-63	40.53 ± 11.46	4.50 ± 1.27
Objective support	9-63	40.36 ± 11.58	4.48 ± 1.29

### 3.3 Descriptive statistics and network predictability

To describe node importance, we reported each node’s mean, standard deviation, EI, BEI, and predictability. Mean predictability was 53.7%, meaning that neighbouring nodes accounted for an average of 53.7% of the variance in each node. Further analysis showed that SSI_12 (“participating in activities together to relieve stress”), SSI_1 (“showing personal concern”), and SSI_7 (“expressing affection”) had the highest predictability (**Table 4**).

**Table 4:** Centrality indices and predictability of network nodes.

Variable	Mean ± sd	Expected influence	Nridge expected influence	Predictability (R²)
SSI_1	4.51 ± 1.66	1.011	0.562	0.602
SSI_2	4.51 ± 1.70	0.802	0.520	0.537
SSI_3	4.54 ± 1.73	0.906	0.577	0.507
SSI_4	4.46 ± 1.64	0.919	0.562	0.565
SSI_5	4.44 ± 1.68	0.970	0.610	0.549
SSI_6	4.51 ± 1.65	0.680	0.157	0.486
SSI_7	4.44 ± 1.67	0.900	0.434	0.602
SSI_8	4.67 ± 1.66	0.921	0.551	0.532
SSI_9	4.46 ± 1.61	0.997	0.427	0.592
SSI_10	4.51 ± 1.62	0.959	0.652	0.552
SSI_11	4.42 ± 1.67	1.014	0.587	0.588
SSI_12	4.54 ± 1.67	1.067	0.604	0.609
SSI_13	4.43 ± 1.68	0.902	0.478	0.561
SSI_14	4.46 ± 1.70	0.794	0.360	0.540
SSI_15	4.55 ± 1.73	0.928	0.607	0.527
SSI_16	4.44 ± 1.69	0.905	0.472	0.518
SSI_17	4.38 ± 1.73	0.878	0.303	0.546
SSI_18	4.62 ± 1.57	0.790	0.383	0.494
CAQ_Baby	2.40 ± 0.80	0.653	-0.098	0.500
CAQ_Pain	2.45 ± 0.76	0.531	-0.093	0.463
CAQ_Control	2.41 ± 0.72	0.467	-0.193	0.522
CAQ_Medical	2.37 ± 0.77	0.656	-0.091	0.474

### 3.4 Network structure

**Figure 1:**
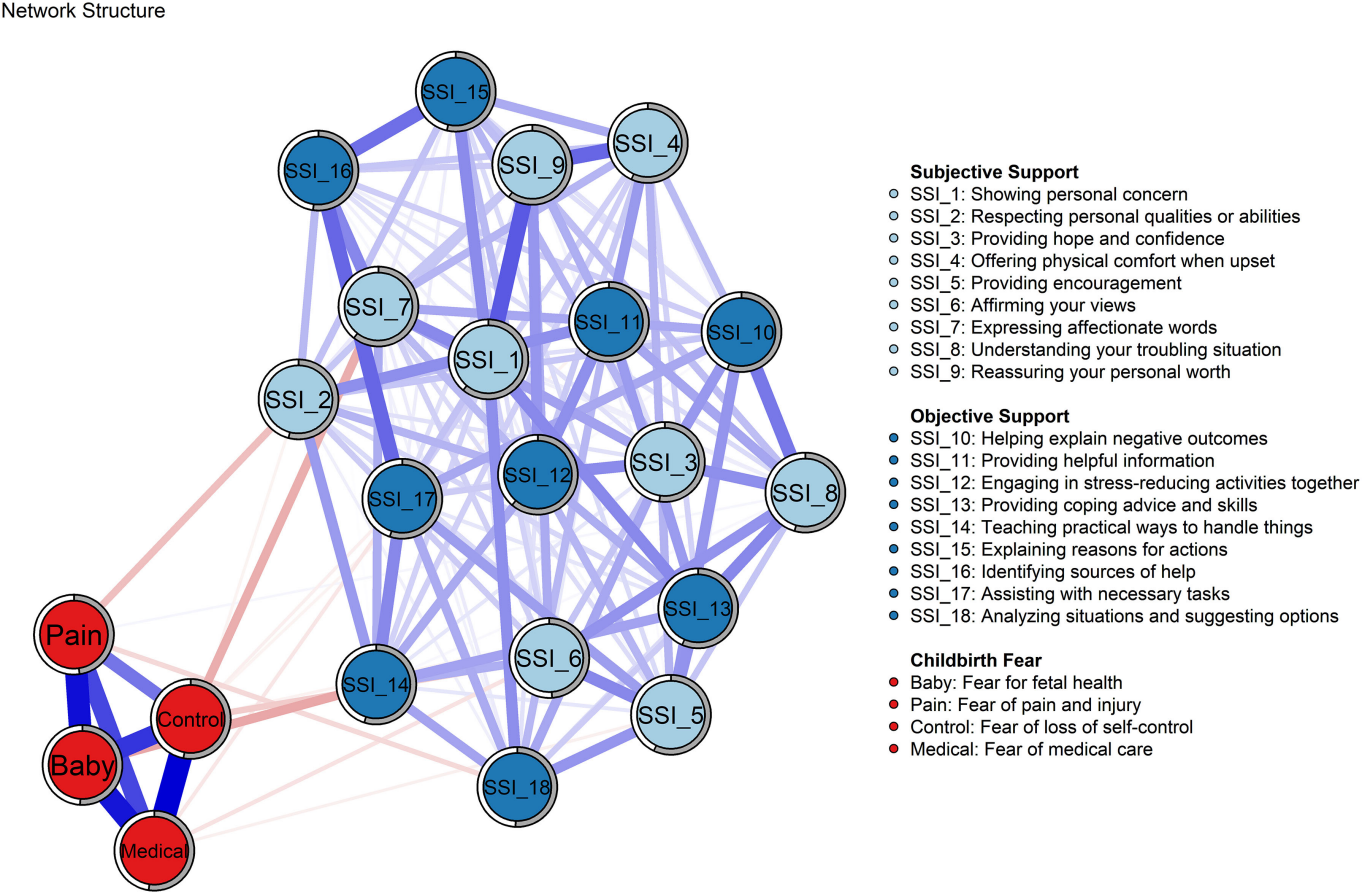
Mixed network of FOC and spousal support. Note: Blue edges indicate positive partial correlations, whereas red edges indicate negative partial correlations. Greater edge thickness or stronger color intensity indicates stronger associations. The filled outer ring of each node represents node predictability; a more fully filled ring indicates higher predictability. Concise descriptions of the node labels are provided in the legend on the right, while their full definitions are detailed in **Supplementary Table 2**.

**Figure 1** presents the 22-node network and the corresponding edge-weight strengths. Of the 231 possible edges, 143 (61.9%) were non-zero, including 131 positive and 12 negative edges. Overall, within-community connections were substantially stronger than between-community connections. Specifically, non-zero intra-community edges within the FOC domain exhibited relatively higher partial correlation coefficients (ranging from 0.15 to 0.28), whereas cross-community associations linking specific spousal supportive behaviors to FOC domains were consistently weak, with absolute edge weights ranging from 0.01 to 0.10. Among cross-community associations, stronger negative edges mainly included SSI_14 (“teaching you how to do things you do not know how to do”) with CAQ_Baby (“concern about fetal health”) (edge weight = −0.098), SSI_7 (“expressing affectionate words to you”) with CAQ_Control (“concern about loss of control”) (edge weight = −0.090), and SSI_2 (“showing respect for personal qualities or abilities”) with CAQ_Pain (“concern about pain”) (edge weight = −0.071). Edge weights are listed in **Supplementary Table 1B**, and bootstrap difference tests for edge weights are shown in **Supplementary Figure 1**.

**Figure 2:**
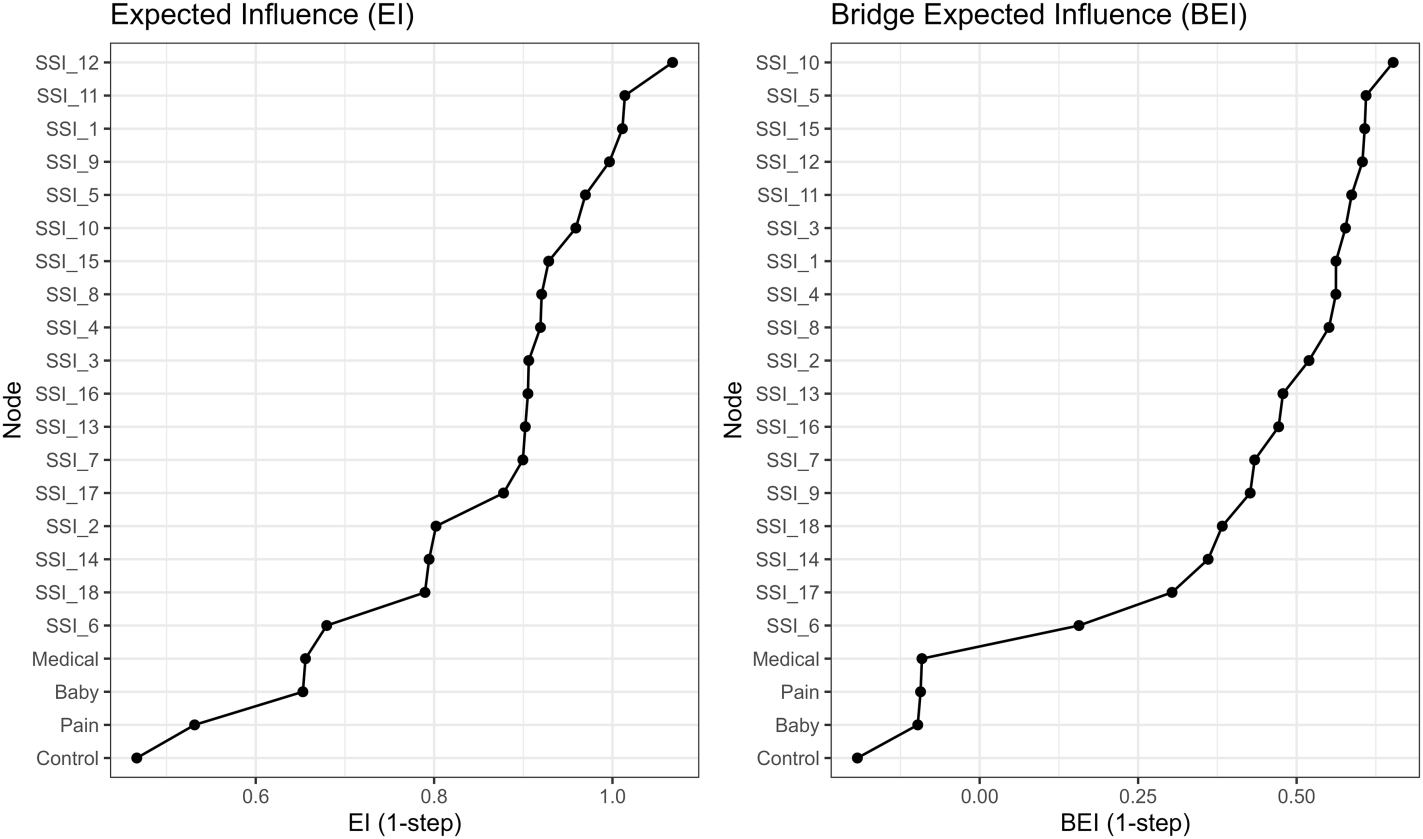
Expected influence and bridge expected influence for FOC and spousal support among pregnant women of advanced maternal age.

The EI and BEI of each node are shown in **Figure 2** (left) and **Figure 2** (right), respectively. In terms of node centrality, the nodes with the highest EI values in the mixed network were SSI_12 (“participating in activities together to reduce your stress”; EI = 1.067), SSI_11 (“providing you with helpful information”; EI = 1.014), and SSI_1 (“showing personal concern”; EI = 1.011). Within the FOC community, “concern about medical intervention” showed the highest influence (EI = 0.656). For bridge centrality, SSI_10 (“helping you understand why things did not go well”) was identified as the most important bridge node linking the spousal support and FOC communities (BEI = 0.652), followed by SSI_5 (“giving you encouragement”; BEI = 0.610) and SSI_15 (“explaining why one should or should not do certain things”; BEI = 0.607). These nodes played key roles in cross-community connectivity.

**Supplementary Figure 2** shows the case-dropping bootstrap results. EI and BEI were both stable, with CS (cor = 0.7) = 0.595. As this value was above the recommended threshold of 0.50, the centrality indices were considered stable even when 59.5% of the sample was randomly removed. **Supplementary Figure 3** presents edge-weight accuracy. Results from the non-parametric bootstrap with 1,000 iterations indicated relatively narrow 95% confidence intervals for edge weights, suggesting high precision of edge weights. In addition, the dimension-level sensitivity network is shown in **Supplementary Figure 4**. In this supplementary 6-node network, subjective and objective spousal support showed a clear positive association, and the four CAQ domains were also interrelated. Cross-construct associations between spousal support and fear of childbirth were present but relatively weak. This pattern was broadly consistent with the primary network, suggesting that the overall association pattern was not solely attributable to the mixed-granularity specification.

## 4 Discussion

In this study, 86.4% of women of advanced maternal age were classified as having any FOC. The CAQ ≥28 threshold was used to maintain comparability with prior CAQ-based studies among Chinese pregnant women and denotes any FOC, encompassing mild (39.8%), moderate (37.3%), and severe (9.3%) categories, rather than only clinically severe fear (5, 22, 25). Thus, although the overall prevalence was high, it should not be interpreted as indicating that most participants experienced pathological fear.

Previous Chinese studies using the CAQ and the same classification criteria reported overall FOC prevalence rates of 67.1% and 67.8%, respectively (5, 22), while a recent large-scale study of women in late pregnancy reported a rate of 79.8% (4). The higher prevalence observed in the present study may be related to specific sample characteristics: participants were exclusively women of advanced maternal age, mostly in late pregnancy, and recruited from a tertiary Grade A hospital, which may limit generalizability. However, our findings align closely with studies focusing specifically on higher-risk or older pregnant women, which reported prevalence estimates of 77.5%–86.3% (26, 27). In addition to sample characteristics, obstetric background provided important clinical context for understanding FOC in this sample. CAQ total scores differed by parity and history of adverse pregnancy outcomes. These findings are broadly consistent with previous evidence suggesting that childbirth fear varies by parity, with higher levels often reported among nulliparous women (28), and that prior adverse obstetric experiences are associated with subsequent fear of childbirth (29).

Notably, although the overall spousal support score was moderately high, the prevalence of FOC remained high. This apparent inconsistency suggests that total support scores alone may obscure clinically meaningful item-level associations and may not adequately reflect how different forms of spousal support relate to specific dimensions of childbirth fear. The effectiveness of support may depend on whether its content and type match women’s specific concerns. Against this background, the present study used network analysis to examine the association structure between spousal support and FOC and to identify specific support elements that may be particularly relevant to psychosocial intervention planning.

Advanced maternal age is associated with increased obstetric risks (6), and women of advanced maternal age may report greater concerns about pregnancy, childbirth, and fetal well-being (4, 5). These factors may make them more sensitive to uncertainty surrounding pregnancy and birth. In this context, specific spousal-support behaviors may not be equally relevant to all domains of childbirth fear. This view is consistent with a recent systematic review and meta-analysis, in which partner support was significantly associated with FOC, whereas family and friend support showed no significant associations (13). Rather than suggesting causal pathways, the present network analysis offers an exploratory approach to identifying which specific support behaviors are more closely associated with particular fear-related concerns. Given the cross-sectional design, the support–FOC associations discussed below should be interpreted as exploratory and hypothesis-generating rather than as confirmed intervention targets. While the cross-community edges were modest in absolute magnitude, they may still provide exploratory information about how specific supportive behaviors are associated with different domains of childbirth fear. The stronger intra-FOC edges may partly reflect the conceptual relatedness of the CAQ domains and their representation as aggregated subscales. Therefore, these findings should be interpreted cautiously and viewed as hypothesis-generating for future studies rather than as direct evidence of intervention targets.

Across the cross-community network, the strongest negative edge was observed between “teaching you how to do things you do not know how to do” (SSI_14) and “concern about fetal health” (CAQ_Baby). This pattern suggests that instrumental support may be particularly relevant to fetal-health-related fear. From the perspective of optimal matching theory, support may be more closely aligned with the recipient’s stress-related needs when its content corresponds to the specific stressor (30).

Centrality analysis further showed that SSI_12 (“participating in activities together to reduce your stress”), SSI_11 (“providing you with helpful information”), and SSI_1 (“showing personal concern”) occupied the most central positions in the mixed network. This pattern suggests that, in women of advanced maternal age, support needs may extend beyond passive companionship and may involve shared activities, informational support, and emotional concern. The prominence of SSI_12 is compatible with Bodenmann’s systemic transactional model of dyadic coping, which emphasizes stress and coping within the couple context (31). In the present network, joint participation in stress-reducing activities may reflect a more active form of partner involvement during pregnancy. Complementing the behavioral engagement represented by SSI_12, the high centrality of SSI_1 and SSI_11 highlights the potential importance of emotional connection and informational support. Accordingly, this dyadic-coping interpretation should be viewed as a theoretical lens rather than evidence of a longitudinal coping process.

Bridge centrality analysis further identified SSI_10 (“helping you understand why things did not go well”), SSI_5 (“giving you encouragement”), and SSI_15 (“explaining why you should or should not do certain things”) as nodes with relatively high bridge expected influence between the spousal support and FOC communities. These nodes share a common feature: they do not simply represent general emotional availability, but reflect appraisal-oriented communication, including helping women understand difficulties, providing encouragement, and offering explanations for decisions or behaviors. This pattern suggests that the connection between partner support and childbirth fear may be particularly evident in communication behaviors that help women organize pregnancy-related uncertainty and interpret childbirth-related concerns. According to interpersonal emotion regulation theory, individuals may draw on close others to help shape their emotional responses to stressful experiences (32). For pregnant women, FOC commonly involves concerns about fetal well-being, labor pain, loss of control, and medical interventions or care (25). In this context, explanation and encouragement from spouses may be especially relevant to how childbirth-related worries are discussed within the couple. Therefore, these bridge nodes may provide useful clues for future studies on couple-based support, particularly regarding how partners communicate about childbirth-related risk, uncertainty, and coping during late pregnancy.

The present findings may have implications for future perinatal mental health research. Rather than treating spousal support as a uniform construct, the network approach highlights specific support behaviors that appear to be more closely connected with FOC domains. In particular, instrumental support, informational support, appraisal-oriented communication, encouragement, and shared stress-reducing activities may provide useful clues for future research on partner involvement among women of advanced maternal age. More broadly, the present focus on partner-related support is consistent with a recent systematic review and meta-analysis showing significant inverse associations between FOC and perceived social support, particularly partner support (13).

Several limitations should be acknowledged. First, the cross-sectional design precludes conclusions about causal direction, temporal order, or dynamic propagation processes. Although bootstrap results indicated acceptable stability, the sample size remained modest for a 22-node psychological network; therefore, the centrality and bridge-centrality findings should be interpreted cautiously and replicated in larger independent samples. Second, while the mixed-level network balances clinical interpretability and robustness, unequal node granularity may influence edge weights. Due to sample constraints, a fully item-level network remains untested, meaning stronger intra-FOC edges might partly reflect the higher shared variance of aggregated domain scores rather than a tighter psychological structure. In addition, communities were assigned *a priori* according to instrument subscales rather than data-driven clustering, which may influence BEI estimates. Third, participants were recruited by convenience sampling from a single tertiary Grade A hospital, and most were in late pregnancy, which may limit the representativeness and generalizability of the findings and should be considered when interpreting FOC levels and spousal-support patterns. Finally, all variables were self-reported, and spousal support was assessed only from the women’s perspective, which limits the dyadic interpretation of partner support; validated measures of antenatal anxiety and depression were also not included. Future multicenter longitudinal studies with larger samples, partner-reported data, and more comprehensive obstetric and psychosocial assessments are needed to validate these findings.

## 5 Conclusion

This study explored the links between spousal support and fear of childbirth among women of advanced maternal age from a network perspective. Different forms of support were associated with childbirth fear in different ways, especially fear related to fetal health. The findings suggest that support from spouses may matter not only at the emotional level, but also in helping women cope with stress, make sense of difficulties, and deal with childbirth-related concerns more effectively. In clinical care, greater attention to practical and cognitively oriented forms of spousal support may help improve support for women of advanced maternal age who experience fear of childbirth.

## Data Availability

The raw data supporting the conclusions of this article will be made available by the authors, without undue reservation.
