# Supplementary material for: Network analysis of spousal support and fear of childbirth in pregnant women of advanced maternal age
Source: Front Psychiatry. 2026 Jun 22;17:1848470. doi: 10.3389/fpsyt.2026.1848470 (PMC13333595; doi:10.3389/fpsyt.2026.1848470)
Supplement: Supplementary file 1 [file SupplementaryFile1.docx]

**Supplementary Table S1A. Ranked cross-community edge weights between spousal support and fear of childbirth.**

| Rank | CSQ node | FOC domain | Edge weight |
| --- | --- | --- | --- |
| 1 | SSI_14 | Baby | -0.10 |
| 2 | SSI_7 | Control | -0.09 |
| 3 | SSI_2 | Pain | -0.07 |
| 4 | SSI_6 | Control | -0.06 |
| 5 | SSI_18 | Pain | -0.04 |
| 6 | SSI_6 | Medical | -0.03 |
| 7 | SSI_17 | Medical | -0.03 |
| 8 | SSI_17 | Control | -0.02 |
| 9 | SSI_5 | Medical | -0.02 |
| 10 | SSI_11 | Control | -0.01 |
| 11 | SSI_13 | Control | -0.01 |
| 12 | SSI_2 | Medical | -0.01 |

**Supplementary Table S1B. Edge weights of all connections in the network of spousal support and fear of childbirth among women of advanced maternal age.**

|  | SSI_1 | SSI_2 | SSI_3 | SSI_4 | SSI_5 | SSI_6 | SSI_7 | SSI_8 | SSI_9 | SSI_10 | SSI_11 | SSI_12 | SSI_13 | SSI_14 | SSI_15 | SSI_16 | SSI_17 | SSI_18 | Baby | Pain | Control | Medical |
| --- | --- | --- | --- | --- | --- | --- | --- | --- | --- | --- | --- | --- | --- | --- | --- | --- | --- | --- | --- | --- | --- | --- |
| SSI_1 | 0.00 |  |  |  |  |  |  |  |  |  |  |  |  |  |  |  |  |  |  |  |  |  |
| SSI_2 | 0.07 | 0.00 |  |  |  |  |  |  |  |  |  |  |  |  |  |  |  |  |  |  |  |  |
| SSI_3 | 0.00 | 0.03 | 0.00 |  |  |  |  |  |  |  |  |  |  |  |  |  |  |  |  |  |  |  |
| SSI_4 | 0.00 | 0.00 | 0.00 | 0.00 |  |  |  |  |  |  |  |  |  |  |  |  |  |  |  |  |  |  |
| SSI_5 | 0.00 | 0.04 | 0.00 | 0.08 | 0.00 |  |  |  |  |  |  |  |  |  |  |  |  |  |  |  |  |  |
| SSI_6 | 0.05 | 0.06 | 0.07 | 0.00 | 0.13 | 0.00 |  |  |  |  |  |  |  |  |  |  |  |  |  |  |  |  |
| SSI_7 | 0.13 | 0.02 | 0.10 | 0.08 | 0.04 | 0.00 | 0.00 |  |  |  |  |  |  |  |  |  |  |  |  |  |  |  |
| SSI_8 | 0.02 | 0.00 | 0.13 | 0.02 | 0.06 | 0.12 | 0.02 | 0.00 |  |  |  |  |  |  |  |  |  |  |  |  |  |  |
| SSI_9 | 0.18 | 0.06 | 0.00 | 0.17 | 0.00 | 0.09 | 0.06 | 0.00 | 0.00 |  |  |  |  |  |  |  |  |  |  |  |  |  |
| SSI_10 | 0.03 | 0.06 | 0.11 | 0.08 | 0.12 | 0.00 | 0.06 | 0.15 | 0.04 | 0.00 |  |  |  |  |  |  |  |  |  |  |  |  |
| SSI_11 | 0.01 | 0.13 | 0.04 | 0.11 | 0.01 | 0.03 | 0.10 | 0.10 | 0.09 | 0.10 | 0.00 |  |  |  |  |  |  |  |  |  |  |  |
| SSI_12 | 0.02 | 0.08 | 0.13 | 0.06 | 0.08 | 0.04 | 0.08 | 0.00 | 0.12 | 0.01 | 0.12 | 0.00 |  |  |  |  |  |  |  |  |  |  |
| SSI_13 | 0.13 | 0.05 | 0.00 | 0.05 | 0.12 | 0.00 | 0.00 | 0.13 | 0.01 | 0.02 | 0.11 | 0.09 | 0.00 |  |  |  |  |  |  |  |  |  |
| SSI_14 | 0.05 | 0.11 | 0.03 | 0.05 | 0.03 | 0.09 | 0.09 | 0.01 | 0.00 | 0.00 | 0.00 | 0.09 | 0.10 | 0.00 |  |  |  |  |  |  |  |  |
| SSI_15 | 0.13 | 0.07 | 0.08 | 0.09 | 0.04 | 0.04 | 0.02 | 0.07 | 0.07 | 0.03 | 0.00 | 0.08 | 0.00 | 0.00 | 0.00 |  |  |  |  |  |  |  |
| SSI_16 | 0.00 | 0.08 | 0.03 | 0.06 | 0.01 | 0.05 | 0.13 | 0.04 | 0.06 | 0.05 | 0.02 | 0.00 | 0.02 | 0.00 | 0.16 | 0.00 |  |  |  |  |  |  |
| SSI_17 | 0.07 | 0.01 | 0.06 | 0.05 | 0.11 | 0.00 | 0.02 | 0.00 | 0.04 | 0.10 | 0.00 | 0.04 | 0.03 | 0.13 | 0.02 | 0.17 | 0.00 |  |  |  |  |  |
| SSI-18 | 0.13 | 0.02 | 0.10 | 0.00 | 0.12 | 0.01 | 0.02 | 0.04 | 0.00 | 0.00 | 0.08 | 0.03 | 0.06 | 0.10 | 0.03 | 0.01 | 0.09 | 0.00 |  |  |  |  |
| Baby | 0.00 | 0.00 | 0.00 | 0.00 | 0.00 | 0.00 | 0.00 | 0.00 | 0.00 | 0.00 | 0.00 | 0.00 | 0.00 | -0.10 | 0.00 | 0.00 | 0.00 | 0.00 | 0.00 |  |  |  |
| Pain | 0.00 | -0.07 | 0.00 | 0.00 | 0.00 | 0.00 | 0.00 | 0.01 | 0.01 | 0.00 | 0.00 | 0.00 | 0.00 | 0.00 | 0.00 | 0.00 | 0.00 | -0.04 | 0.27 | 0.00 |  |  |
| Control | 0.00 | 0.00 | 0.00 | 0.00 | 0.00 | -0.06 | -0.09 | 0.00 | 0.00 | 0.00 | -0.01 | 0.00 | -0.01 | 0.00 | 0.00 | 0.00 | -0.02 | 0.00 | 0.22 | 0.15 | 0.00 |  |
| Medical | 0.00 | -0.01 | 0.00 | 0.00 | -0.02 | -0.03 | 0.00 | 0.00 | 0.00 | 0.00 | 0.00 | 0.00 | 0.00 | 0.00 | 0.00 | 0.00 | -0.03 | 0.00 | 0.26 | 0.20 | 0.28 | 0.00 |

**Supplementary Table S2. Node codes and item descriptions used in the network analysis.**

| node | item description |
| --- | --- |
| SSI_1 | showing personal concern |
| SSI_2 | showing respect for personal qualities or abilities |
| SSI_3 | giving you hope or confidence |
| SSI_4 | comforting you through physical actions when you are upset |
| SSI_5 | giving you encouragement |
| SSI_6 | affirming your views in many situations |
| SSI_7 | expressing affectionate words to you |
| SSI_8 | showing understanding of the situation that troubles you |
| SSI_9 | telling you that you are still a good person even when you encounter difficulties |
| SSI_10 | helping you understand why things did not go well |
| SSI_11 | providing you with helpful information |
| SSI_12 | participating in activities together to reduce your stress |
| SSI_13 | providing advice on how to do things or informing you of the skills needed to cope with the situation |
| SSI_14 | teaching you how to do things you do not know how to do |
| SSI_15 | explaining why you should or should not do certain things |
| SSI_16 | informing you about who can provide help or assistance |
| SSI_17 | assisting you with tasks that need to be done |
| SSI_18 | analyzing the situation with you and suggesting feasible options |
| CAQ_Baby | fear for fetal health |
| CAQ_Pain | fear of pain and injury |
| CAQ_Control | fear of loss of self-control |
| CAQ_Medical | fear of medical care |

**Supplementary Table S3. Fear of childbirth across selected obstetric and clinical characteristics.**

| variable | Category | n (%) | Any FOC, n (%) | CAQ total score, mean ± SD | P value |
| --- | --- | --- | --- | --- | --- |
| Current gestational age (weeks) | 28-32 | 37 (13.3) | 32 (86.5) | 37.78 ± 9.64 | 0.873 |
|  | 33-36 | 169 (60.6) | 144 (85.2) | 38.52 ± 10.56 |  |
|  | ≥37 | 73 (26.2) | 65 (89.0) | 38.84 ± 8.78 |  |
| Parity | Nulliparous | 150 (53.8) | 133 (88.7) | 39.72 ± 10.13 | 0.028 |
|  | Multiparous | 129 (46.2) | 108 (83.7) | 37.09 ± 9.64 |  |
| Planned pregnancy | Yes | 191 (68.5) | 167 (87.4) | 39.19 ± 9.90 | 0.089 |
|  | No | 88 (31.5) | 74 (84.1) | 37.01 ± 10.02 |  |
| History of adverse pregnancy outcomes | Yes | 70 (25.1) | 56 (80.0) | 39.24 ± 9.96 | 0.032 |
|  | No | 209 (74.9) | 185 (88.5) | 36.30 ± 9.74 |  |
| Use of tocolytic medication during pregnancy | Yes | 93 (33.3) | 86 (92.5) | 39.77 ± 8.39 | 0.133 |
|  | No | 186 (66.7) | 155 (83.3) | 37.87 ± 10.64 |  |
| Preferred mode of childbirth | Vaginal birth | 161 (57.7) | 137 (85.1) | 38.52 ± 9.88 | 0.978 |
|  | Cesarean section | 47 (16.8) | 40 (85.1) | 38.26 ± 10.35 |  |
|  | No strong preference | 71 (25.4) | 64 (90.1) | 38.65 ± 10.08 |  |
| Intention to use labor analgesia | Yes | 249 (89.2) | 216 (86.7) | 38.68 ± 9.88 | 0.404 |
|  | No | 30 (10.8) | 25 (83.3) | 37.07 ± 10.81 |  |
| Pregnancy comorbidities or complications | Yes | 84 (30.1) | 72 (85.7) | 38.82 ± 9.75 | 0.729 |
|  | No | 195 (69.9) | 169 (86.7) | 38.37 ± 10.09 |  |

**Note. CAQ, Childbirth Attitude Questionnaire; FOC, fear of childbirth. Any FOC was defined as a CAQ total score ≥28. Percentages are calculated within each category. P values were obtained from comparisons of CAQ total scores using independent-samples t tests or one-way ANOVA, as appropriate**


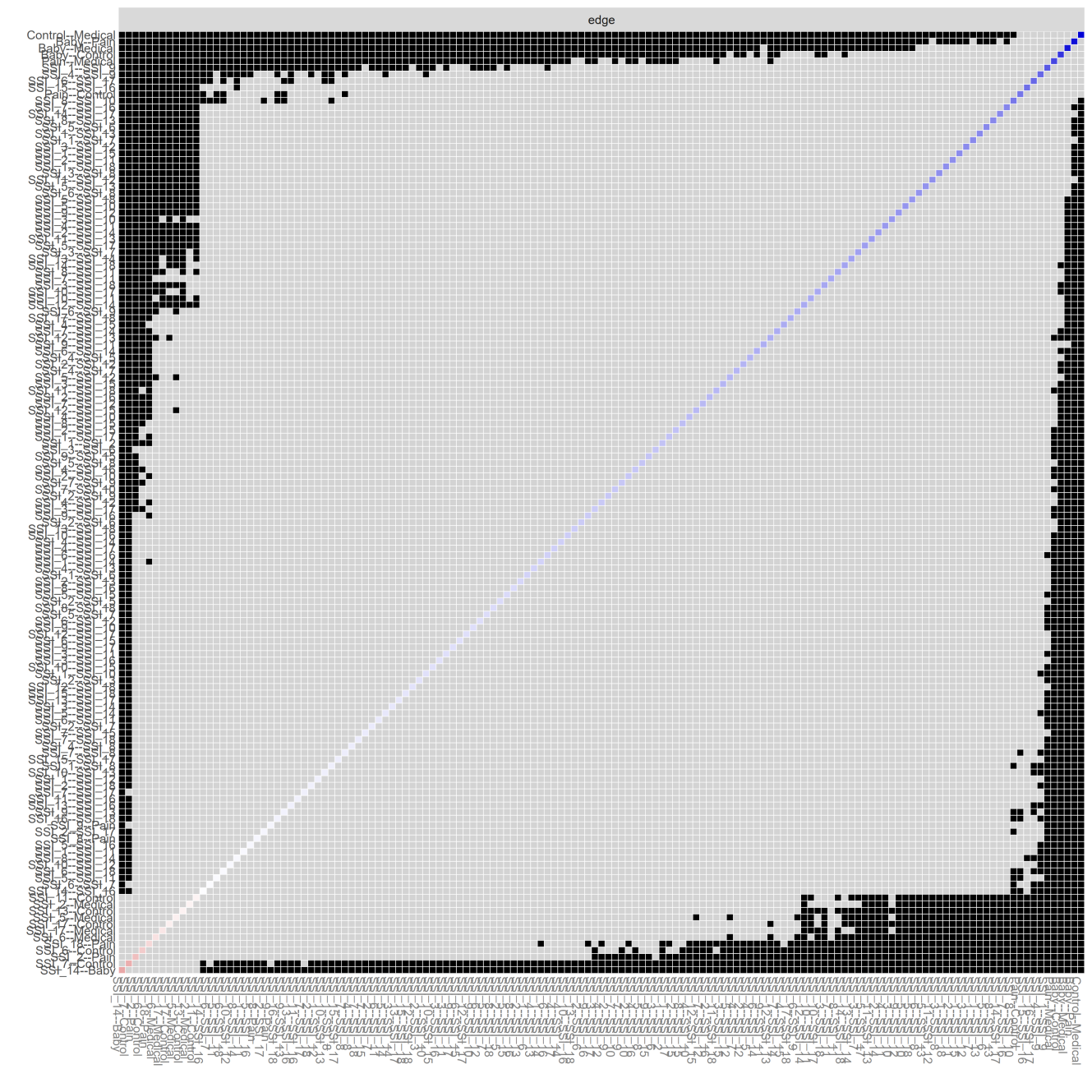


**Supplementary Figure S1. Bootstrap difference test of edge weights in the network structure of spousal support and fear of childbirth among pregnant women of advanced maternal age**

**Note: Gray boxes indicate no significant difference in edge weights, while black boxes denote a significant difference. The blue and red boxes along the diagonal correspond to positively and negatively correlated edge weights, respectively**


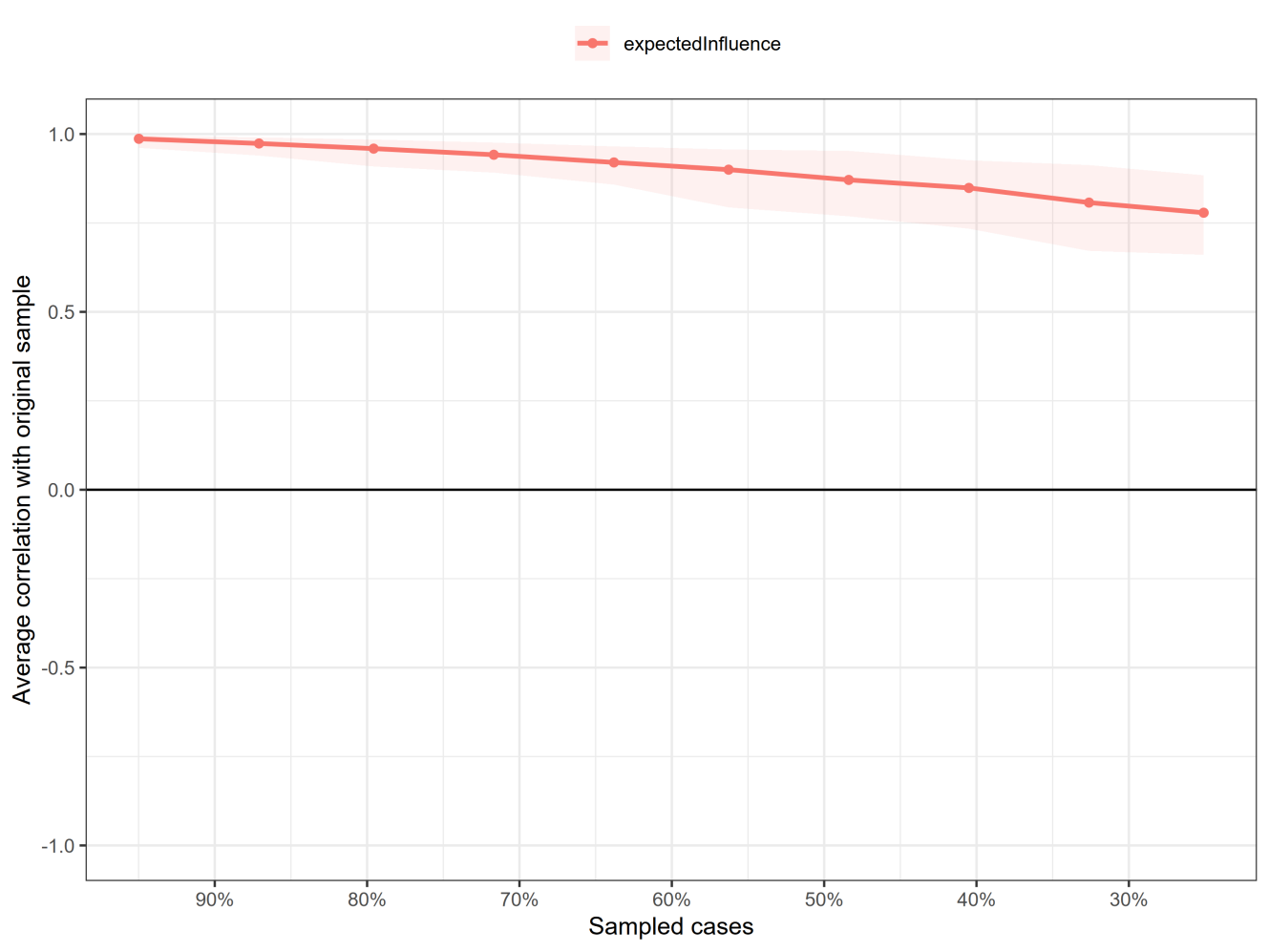


**
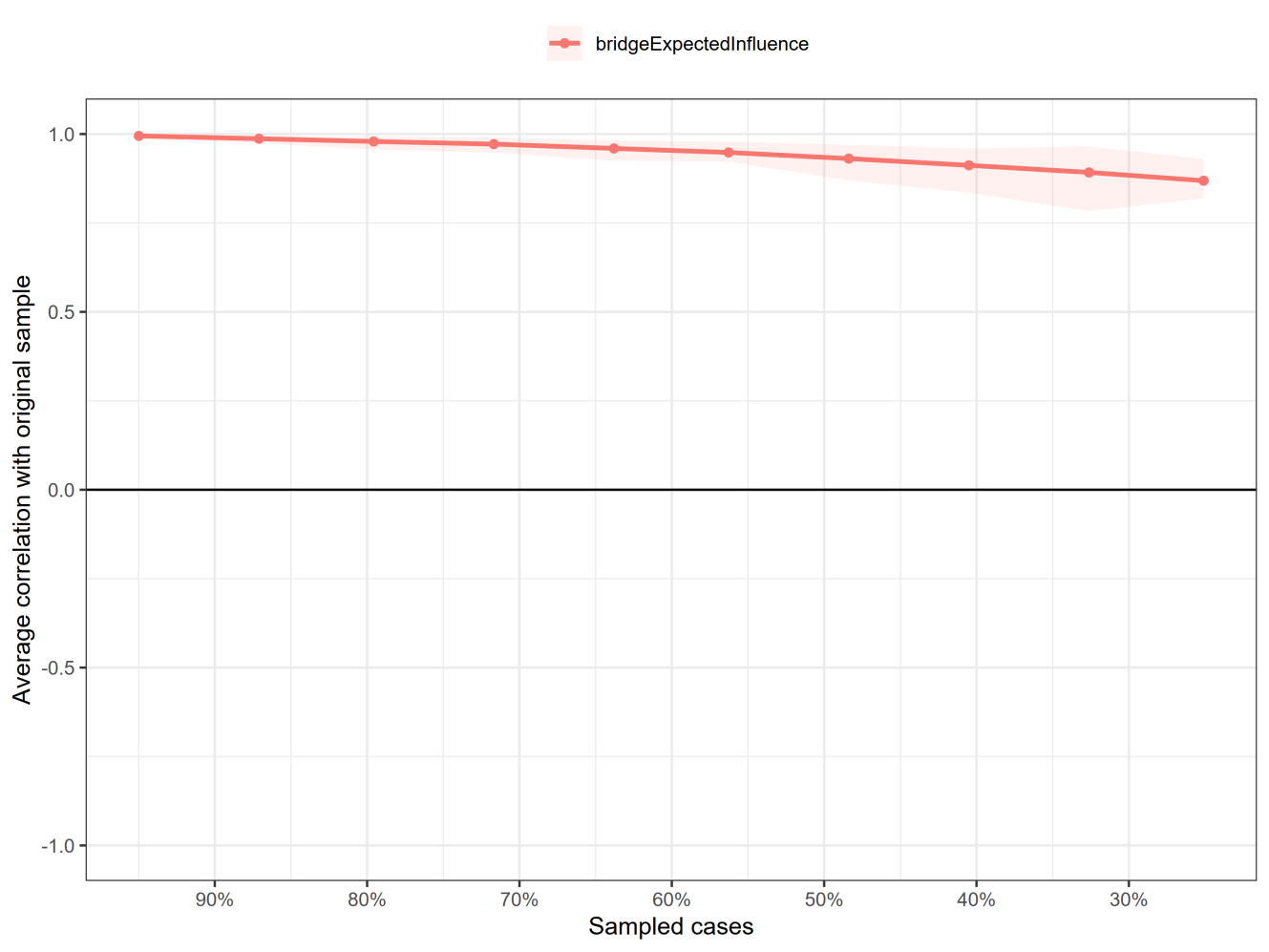
**

**Supplementary Figure S2. Stability test of expected influence and bridge expected influence**

**
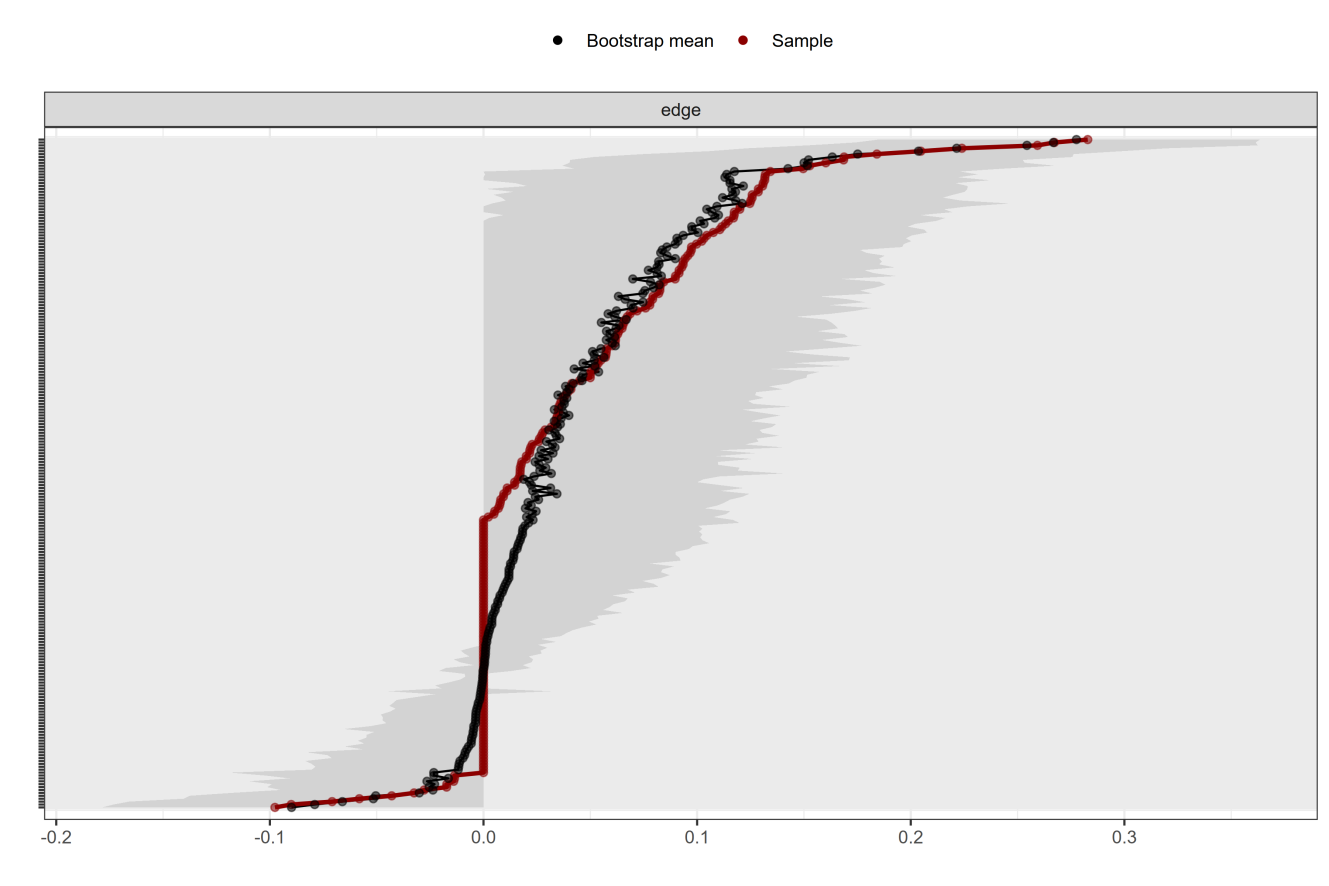
**

**Supplementary Figure S3. Accuracy of edge weights in the network of spousal support and fear of childbirth**

**
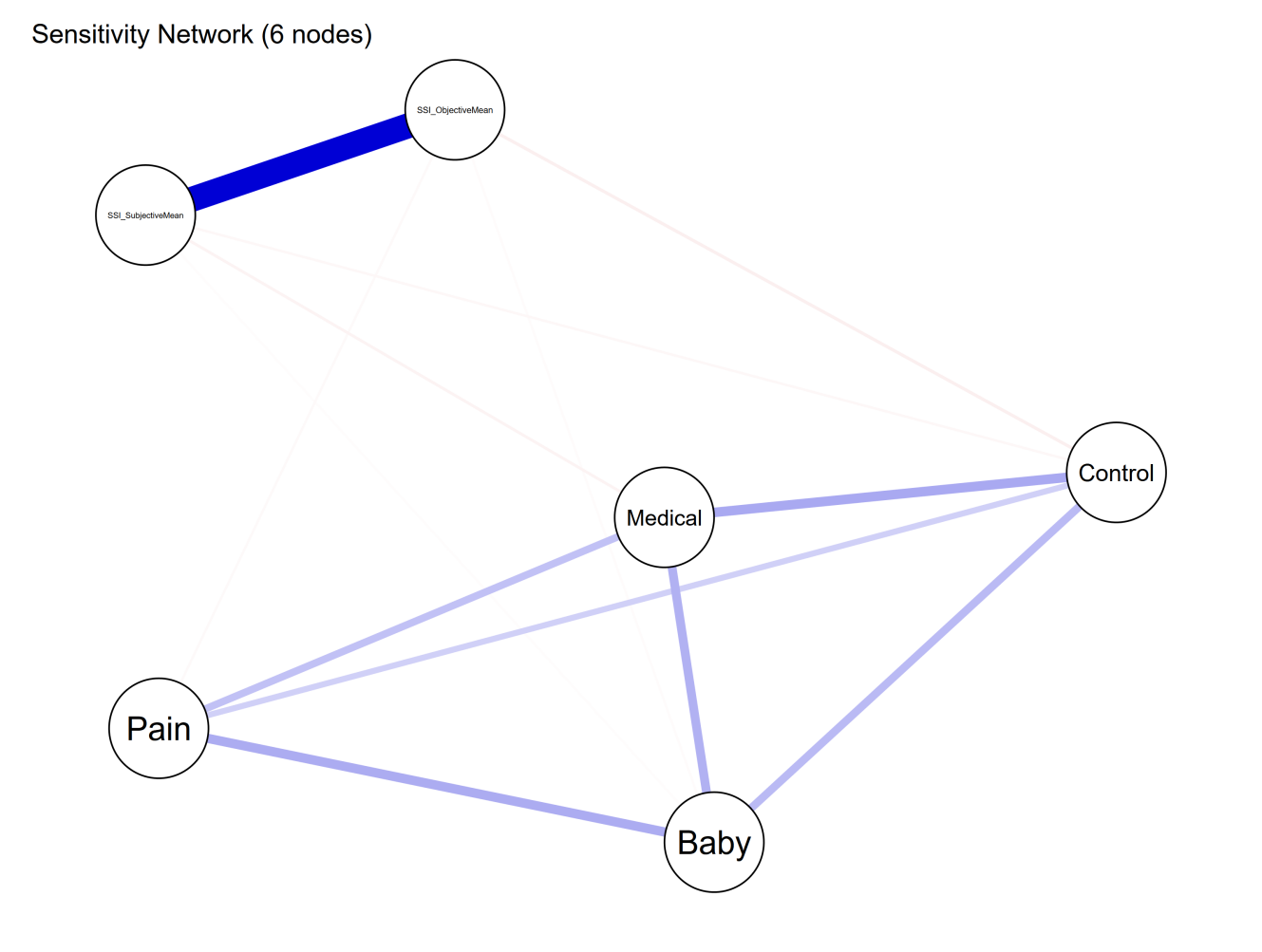
**

**Supplementary Figure S4. Dimension-level sensitivity network of spousal support and fear of childbirth**
